# Glances at the Insane in Other Lands

**Published:** 1889-01-12

**Authors:** 


### Glances at the Insane in Other Lands.

By a Medical Superintendent.

*(Continued from p. 164.)*

### III.—PENNSYLVANIA.

(Fifth Report of the State Committee on Lunacy).

There is one thing in which we must yield the palm to our American cousins, and that is the admirable manner in which their State documents are arranged. There is a clearness and a directness about their work in these matters with which we in the old country try in vain to cope. Contrast the report whose title stands at the head of this article with one of our Blue-books, and it must be evident to all men that the inferiority of the latter is most striking, whether we compare the type, the paper, or the letterpress.

We find on the fly-leaf of the report which we are about to notice a coloured map of the State, showing the manner in which it is divided into districts, and further on there is another map showing the parts from which the asylum at Harrisburg draws its patients. Other maps of the sub-districts follow in quick succession, so that it is an easy matter even for a stranger to follow the descriptions. Then we have plans of the Cane Hill Asylum, of the York Retreat, of Dr. Clouston's Model Asylum for 200 patients, of the Asylum designed by Dr. Greene, of the Rittergut Asylum, Saxony; of the Pilsen Asylum, Austria; of the Asylum at Lyons, and of the Bron Asylum, France. Full descriptive letterpress of many of these is given.

Pennsylvania was the State which took the lead in America in providing proper accommodation for its insane patients, and the authors of this report point out that the humane treatment inaugurated by Pinel in Europe was anticipated nearly fifty years by the framers of the charter for the Hospital at Philadelphia, which dates back to 1750. It is, however, admitted that the condition of the insane was not what it ought to have been for many years, and it was not until 1845 that steps were taken to build the State Hospital at Harrisburg. It will show how things have grown in the United States, as with us, when we mention that up to the present time Pennsylvania has expended, in equipping and maintaining its asylums, a sum exceeding six and a-quarter million dollars.

Not fewer than 19,191 patients have been admitted to the various lunatic hospitals, and of these 7,771 have recovered; 3,471 are stationary; 3,714 have died; and 13 were discharged as not insane. At present 4,222 are under treatment.

The question of providing a separate asylum for criminal unatics has been mooted, and it is stated that the gaols and lunatic hospitals of the State contain already 227 of that class. All of these would not be suitable cases for removal to a criminal asylum, and the State Committee say that even if they were so removed, the beds thereby obtained would not form any appreciable addition to the existing hospitals. But surely this is not the way to look at it. The advantage of freeing the ordinary lunatics from association with criminals is so great and so evident that it need hardly be insisted on.

Provision for the middle-class insane in Pennsylvania would seem to be in as backward a state as it is in England. Owing to want of room, we are told, such cases have been debarred admission, and have been obliged either to remain at home, or apply to private institutions or enter an almshouse, or be sent direct to a hospital by order of court. As this is exactly what happens in England, we need not offer much comment on it. The following paragraphs are so true and so much to the point, that we make no apology for quoting them verbatim : "The middle class is, like the average indigent insane were prior to their mental affliction, a self-supporting and self-respecting element in the community, and, so long as not overtaken by insanity, are fully able to care for themselves ; but when disabled by sickness, their limited resources often debar them from securing the costly treatment in private institutions."

"The insane of the middle class, now that the indigent have all been provided for, should be admitted into our State institutions on payment of at least the actual cost of maintenance, or possibly at a less rate, in the discretion of the hospital management ; especially should those acute cases be received which are usually promptly restored by appropriate treatment. There ought to be no question on this point."

The new world, in spite of all its resources, is cursed with the same increase in the numbers of its registered insane as the old countries of Europe. In 1840 Pennsylvania had 2,133 insane persons. In 1880 the numbers had increased to 6,961, and while the population of the State had increased from 1,724,000 to 4,242,000 the per centage of the insane had increased from one-eleventh, to one-seventh.

Harrisburg State Lunatic Hospital: This is the hospital for the southern district of the State. The district contains about one million inhabitants, and it has an area of 10,468 square miles. The hospital was opened in 1851, large additions have lately been completed, and its capacity is now 732 beds. The cost of maintenance is about 15s. 1d. per head per week. We are pleased to learn that no male patient was subjected to mechanical restraint during the year. Three females were restrained, but all for surgical reasons. One hundred and fifty patients were admitted during the year ; 31 were discharged recovered, 24 relieved, and 44 died.

In addition to the medical superintendent, there are four medical officers, and two of these are ladies. The appointment of lady medicals is very common in American asylums, the system is said to answer admirably. It is strange that so manifest an improvement has not yet been introduced into our asylums. The attendants are in the proportion of one attendant to nine patients, a proportion somewhat higher than pertains in England.

The Dixmont Hospital provides for the wants of the south-western district. It contains 470 beds. The weekly cost of maintenance is just a trifle lower than in the hospital last noticed. There are in all four medical officers. We see that that the office of matron has been abolished, and that the system of head nurse and housekeeper has been substituted. During the year 208 patients were admitted. There were 195 discharges and 77 deaths. The instances of restraint were somewhat numerous, at least they sound so in English ears, although it is said to be used only when absolutely necessary.

The hospital for the northern district is situated at Danville. The population of the district is 800,000, and there is an area of 14,887 square miles. The hospital can properly accommodate only 700 patients, but it contained 794 at the close of the year, 200 of these not belonging to the district.

The average cost per head per week is about 14s. 7d. The hospital is superintended by Dr. Schultz, and he has three assistant-physicians. The average daily number of patients resident was 848. The recoveries calculated on this basis were 5·31 and the deaths 6·13 per cent. Here, again, the amount of restraint strikes us as large ; but without being acquainted with the individual cases it is unfair to make any adverse comment. Fifteen men and twelve women were restrained, the reasons being chiefly destructive habits or suicidal tendencies.

The hospital at Norristown draws its patients from the south-eastern part of the state. The district has a population over a million and a-quarter, and includes an area of 2,913 square miles. The hospital is the largest in the state, and can accommodate 1,600 patients. The trustees suggest its enlargement by 200 beds, a step which is of more than doubtful policy.

The average cost per head per week is 14s. 7d.

There are nine medical officers, and four of these are ladies. The attendants are in the proportion of one attendant to eleven patients. Four hundred and sixty-eight patients were admitted during the year. The average daily number resident was 1,508. The deaths calculated on this were 10·28 per cent., and the recoveries were 5 per cent., calculated on the same basis. Considerably over 50 per cent. of the men employ themselves, and nearly the same proportion of the women are engaged in useful occupations of some kind. During the year several male patients have been restrained by mild mechanical means for excitement, violence, or destructiveness. No female was in restraint during the year.

Warren Hospital for the North-Western District.—The average number of patients here was 676, being an increase of 39 on the former year. The admissions were 222. The deaths were 10 per cent. on the average number resident, and the recoveries calculated in the same way were 7·54 per cent. A mild form of mechanical restraint is occasionally used. There is one medical superintendent, and two assistant medical officers.

The Hospital at Philadelphia is the oldest institution for the insane in the United States, and it dates back to 1751. Last year the average number resident was 408, showing an increase of 36 on the previous year. The deaths were 7·60 per cent., calculated on the above number, and the recoveries were 11 per cent. The library contains 2,700 volumes. The average weekly cost per head per week is £1 16s. 3d. The proportion of attendants is very high, and there are four assistant medical officers. Restraint was used in the cases of 7 men and 20 women—the causes being given as homicidal attacks, destructiveness, surgical reasons, suicidal tendencies, etc. This amount of restraint seems high, when we look at the number of patients and the proportion of attendants.

The General Hospital at Philadelphia has an insane department, containing 600 patients, and there are several small private asylums in the state.

### SCHOOL OF MASSAGE AND ELECTRICITY.

The next session of the School of Massage and Electricity in connexion with the West End Hospital, 73, Welbeck Street, London, will commence on January 14th inst. A full course of lectures is given at this institution, with practical tuition. It is understood that many of the pupils find employment both in connexion with the lectures and as the result of their training and association with the hospital.

